# Albumin as a Prognostic Indicator in Pre-Intubated Patients With SARS-CoV-2: A Retrospective, Multi-Institutional Study

**DOI:** 10.7759/cureus.18532

**Published:** 2021-10-06

**Authors:** Kritika Subramanian, Gary Brandeis

**Affiliations:** 1 Molecular Imaging and Therapeutics, Weill Cornell Medicine, New York, USA; 2 Internal Medicine, Icahn School of Medicine at Mount Sinai, Elmhurst, USA

**Keywords:** sars-cov-2, biomarker, pre-intubation, nippv, serum albumin

## Abstract

Background

Much effort has been placed on evaluating serological tests that can predict worsening prognosis in severe acute respiratory syndrome coronavirus-2 (SARS-CoV-2) infection. Endotracheal intubation in SARS-CoV-2 is associated with a higher risk of mortality. While studies have evaluated serological markers that can predict worsening prognosis, the likelihood of intubation in these patients has not been evaluated. The objective of this study was to determine if any serum marker corresponded to oxygen escalation or de-escalation in SARS-CoV-2-infected pre-intubated patients.

Methodology

This retrospective study reviewed 1,754 SARS-CoV-2 patients in the New York City Health and Hospitals Corporation (NYCHHC) system who required non-invasive positive pressure ventilation (NIPPV) such as continuous positive airway pressure or bilevel positive airway pressure. All patients were above the age of 18, were not terminally ill and requiring hospice care, and were admitted to the NYCHHC system between March 1, 2020 and May 17, 2020. SARS-CoV-2 serological labs were collected for five days for patients initiated on NIPPV, such that day one was 24 hours after NIPPV initiation.

Results

Multivariate and univariate linear regression modeling on this population cohort was remarkable for a significant association between serum albumin levels and oxygen escalation or de-escalation from NIPPV.

Conclusions

We conclude that serum albumin level may have further utility in predicting oxygen escalation in pre-intubated patients with SARS-CoV-2, especially in a low-resource and high-demand setting.

## Introduction

In light of increasing morbidity and mortality from severe acute respiratory syndrome coronavirus-2 (SARS-CoV-2) infection, much effort has been placed on evaluating serological biomarkers that can predict worsening prognosis. Post-anticoagulant D-dimer was shown to predict in-hospital mortality in a cohort of 1,835 adult patients with polymerase chain reaction-confirmed SARS-CoV-2 [1]. Topp et al. (2021) demonstrated that patients who survived the infection had lower D-dimer, ferritin, lactate dehydrogenase (LDH), and aspartate aminotransferase (AST) levels when they were intubated [2]. Alternatively, a multivariate model aimed at identifying the risk for poor outcomes applied on a sample cohort of 2,545 patients in New York City found that age, albumin, creatinine, C-reactive protein (CRP), and LDH were predictive variables [3]. Identifying at-risk patients is crucial for providing care and determining resource allocation based on patient risk stratification. Endotracheal intubation in SARS-CoV-2 has been associated with a higher risk of mortality [4], and determining an efficient way of identifying patients who are likely to require intubation would assist in providing enhanced care and prioritizing resources in a high-demand and limited-resource setting [5].

As New York City was one of the epicenters at the beginning of the pandemic in the United States, a convenient “COVID panel” including multiple serological tests was adopted as an institutional analytical tool for all patients admitted to the public hospital system known as the New York Health and Hospitals Corporation (NYCHHC). These tests were requested at the discretion of the primary care provider(s). In addition, various interventions were attempted, especially regarding the need for oxygenation. These included nasal cannula, face mask oxygen, continuous positive airway pressure (CPAP), and bilevel positive airway pressure (BiPAP) before intubation and ventilation. At the time, high-flow oxygen was not being used due to the concern of excess aerosolization of the virus as an institutional policy. Because patients placed on non-invasive positive pressure ventilation (NIPPV) such as CPAP or BiPAP indicated an escalation of their care, there were questions regarding whether serum markers could predict further escalation (i.e., mechanical ventilation).

Measuring oxygen escalation and de-escalation from the midpoint of oxygen requirements simplified the analysis, as what constituted escalation and de-escalation could be easily defined. Escalation of care from NIPPV was defined as intubation while de-escalation was to any form of supplemental oxygen which was not NIPPV or endotracheal intubation. The primary objective of this study was to examine the “COVID panel” lab values at the time of NIPPV initiation to ascertain if any serum lab parameter was predictive of the outcome of oxygenation requirements. We hypothesized that labs which corresponded to inflammation such as albumin, CRP, and erythrocyte sedimentation rate (ESR) were predictive of escalation or de-escalation of oxygen requirements in SARS-CoV-2 patients who were on NIPPV.

## Materials and methods

This retrospective study reviewed SARS-CoV-2 patients in the NYCHHC system who were admitted between March 1, 2020 and May 17, 2020. The inclusion criteria included patients aged over 18 years, those not terminally ill and requiring hospice care, those requiring the assistance of NIPPV during the hospital admission, and those confirmed to have SARS-CoV-2 infection using serological testing of immunoglobulin M (IgM) antibodies and/or radiologically using a CT chest. The exclusion criteria included pediatric and terminally ill individuals, patients who were admitted primarily for a non-SARS-CoV-2 indication, and those who did not require NIPPV during the time of their admission. The 11 hospitals included in this review are Bellevue Hospital, Coney Island Hospital, Elmhurst Hospital, Harlem Hospital, Jacobi Hospital, Kings County Hospital, Lincoln Hospital, Metropolitan Hospital, North Central Bronx Hospital, Queens Hospital, and Woodhull Hospital. Together, these 11 hospitals form the largest public hospital network in the United States.

Serological tests were collected for five days in patients initiated on NIPPV, such that day one was 24 hours after NIPPV initiation. Table 1 lists the serological labs that were obtained for the patients and their normal ranges. The serological labs extracted were based on a standardized ordering set of serological labs called the “COVID panel” which was collected for all patients admitted within the NYCHHC system during the first surge on a mostly daily basis. The extracted data were de-identified.

**Table 1:** COVID panel labs extracted for pre-intubated patients infected with SARS-CoV-2. COVID: coronavirus disease; SARS-CoV-2: severe acute respiratory syndrome coronavirus-2

Serology marker	Normal range
White blood cell	4.5–11.0 × 10^9^/L
Procalcitonin	0.10–0.49 ng/mL
D-dimer	<0.4 µg/mL
Lactate dehydrogenase	140–280 U/L
C-reactive protein	0.8–1.0 mg/dL
Alanine aminotransferase	7–55 U/L
Aspartate aminotransferase	8–48 U/L
Creatinine	0.5–1.2 mg/dL
Platelet count	150,000–450,000/µL
Absolute lymphocyte count	1,000–4,800/µL
Albumin	3.5–5.5 g/dL
Lactate	4.5–19.8 mg/dL

A data collection sheet and a data dictionary were prepared to extract data for all SARS-CoV-2 patients who required NIPPV, met our inclusion criteria, and were admitted in the NYCHHC system between March 1, 2020 and May 17, 2020. This resulted in a total of 1,974 patients. Demographic information including age, gender, body mass index (BMI), and Charlson Comorbidity Index was also extracted.

Statistical analysis was performed using a univariate and multivariate linear regression model. Serological tests over five days were selected based on the understanding that the average duration of NIPPV usage in a small cohort of patients with acute respiratory failure from severe acute respiratory syndrome was 84.3 hours [6].

Oxygen changes were defined as escalation if the patient was intubated before or by day five of evaluation. Oxygen de-escalation was defined as the transition to a source of oxygen that required less oxygen supply such as a non-rebreather mask, nasal cannula, or room air while escalation was identified as intubation. The starting point for the evaluation of oxygen escalation and de-escalation in this study was NIPPV. Patients who were on the nasal cannula or a non-rebreather mask and required escalation to NIPPV were not included in this analysis to maintain consistency in the starting point of evaluation. Stable patients were those who had no change in their oxygen requirements during the five days of extracted data.

The study protocol was approved by the Biomedical Research Alliance of New York (BRANY) via the NYCHHC institutional review board and sponsored by NYCHHC’s Department of Population Health.

## Results

Patient demographics

The average age among the 1,974 patients was 66 years. A total of 1,134 patients were male. The average BMI of patients was 30. A total of 1,607 patients had a Charlson Comorbidity Score of 0. Table 2 further summarizes the patient demographics.

**Table 2:** Summary of patient demographics. BMI: body mass index

n = 1,974		
Age	18–30 years	37
	31–40 years	91
	41–50 years	186
	51–60 years	364
	61–70 years	514
	71–80 years	423
	81–90 years	298
	>91 years	61
Gender	Male	1,134
	Female	840
BMI	Normal (<25)	549
	Overweight (25–30)	523
	Obese (30–35)	346
	Morbidly obese (>35)	448
Charlson Comorbidity Index	0	1,607
	1	364
	2	3

Linear regression models

First, variables with over 10% of missing data were removed. Subsequently, patients who had missing data in any of the evaluated variables were excluded. This resulted in a total sample of 1,754 patients as LDH, CRP, procalcitonin, absolute lymphocyte count, and D-dimer were removed from the analysis because these were not routinely collected within the NYCHHC system. Univariate analysis was performed to evaluate if the individual serological tests may have a causal relationship with oxygen changes. This analysis showed significance for serum creatinine (p < 0.001), platelet count (p = 0.0291), and albumin (p < 0.001) levels. Multivariate linear regression modeling was remarkable for a significant association between oxygen changes and serum creatinine, procalcitonin, and albumin levels (Table 3). All values were rounded to three significant figures.

**Table 3:** Multivariate linear regression modeling for serological variables with no more than 10% of missing data. ANOVA: analysis of variance; df: degree of freedom; SS: sum of square; MS: mean square; WBC: white blood cell; ALT: alanine aminotransferase; AST: aspartate aminotransferase; PLT: platelet count

ANOVA						
	df	SS	MS	F	Significance F	
Regression	7	459.166	65.595	7.121	<0.001	
Residual	1,745	16,075.032	9.212			
Total	1,752	16,534.199				
	Coefficients	Standard error	t Stat	P-value	Lower 95%	Upper 95%
Intercept	-1.297	0.331	-3.923	<0.001	-1.946	-0.649
WBC	-0.025	0.013	-1.929	0.054	-0.05	0
ALT	0	0.001	-0.218	0.828	-0.003	0.002
Creatinine	-0.132	0.03	-4.441	<0.001	-0.191	-0.074
AST	0	0.001	0.002	0.999	-0.002	0.002
PLT	0.001	0.001	2.054	0.04	0	0.003
Albumin	-0.335	0.071	-4.716	<0.001	-0.474	-0.196
Lactate	0.043	0.034	1.258	0.209	-0.024	0.11

Correlating albumin with oxygen changes

Patients with an initial albumin level greater than 3.5 g/dL were significantly more likely to have a de-escalation of their oxygen requirements (p < 0.001). Overall, 75% of patients with an initial serum albumin level greater than 3.5 g/dL had stable or de-escalated oxygen requirements, while 25% of patients required an escalation of their oxygen requirements. This was in comparison to 38% of patients with a serum albumin level less than or equal to 3.5 g/dL who had an escalation of their oxygen requirements. Hence, serum albumin level was an independent predictor for oxygen escalation or de-escalation status.

**Figure 1:**
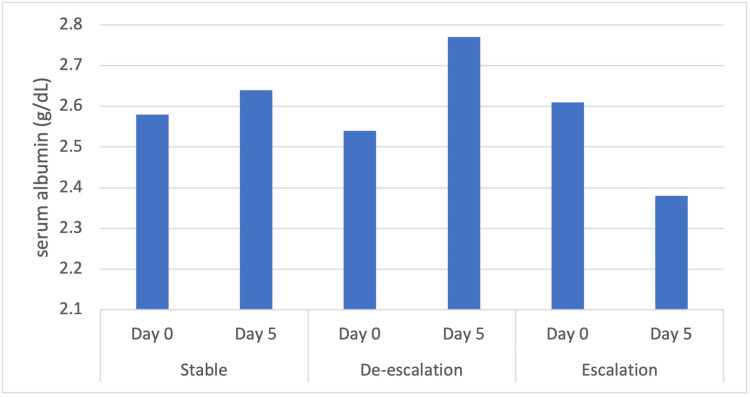
Serum albumin trend for patients with a below normal initial serum albumin level shows that a worsening serum albumin trend was noted in patients who had an escalation of their oxygen requirements.

The average initial serum albumin level for the escalation cohort group was 3.2 g/dL, the de-escalation cohort had an average serum albumin level of 3.5 g/dL, and the stable cohort had an average serum albumin level of 3.3 g/dL. The average change in serum albumin level in the escalation cohort was a decrease of 0.83 g/dL. Figure 1 demonstrates that in patients with below normal serum albumin levels (<3.5 g/dL) at the time of NIPPV initiation, an overall decrease in serum albumin levels corresponded to the escalation of oxygen, while an overall increase in serum albumin levels corresponded to stable or de-escalation of oxygen.

Correlating creatinine with oxygen changes

**Figure 2:**
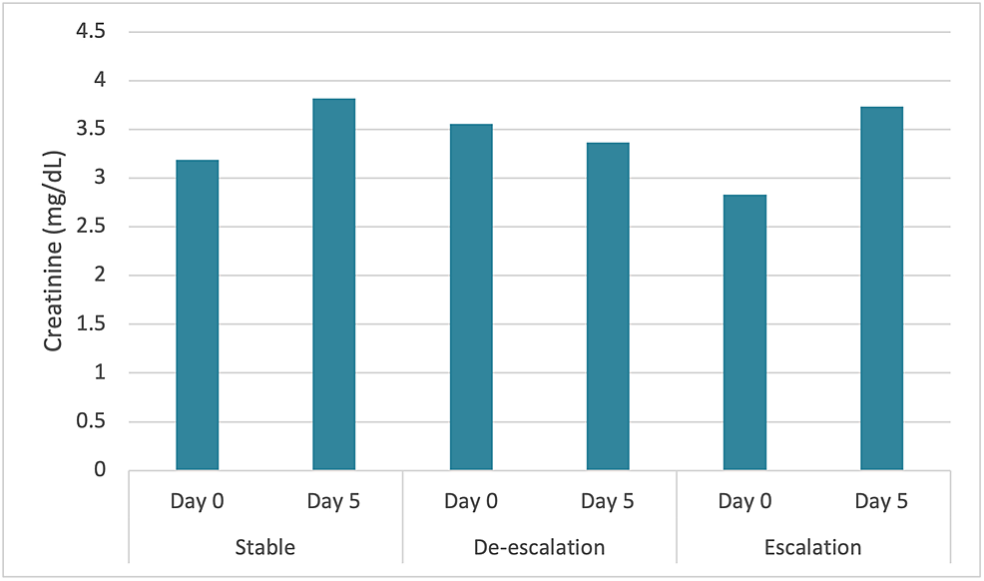
Serum creatinine trends in patients who had above normal creatinine levels at the time of NIPPV initiation. NIPPV: non-invasive positive pressure ventilation

Patients who had an initial serum creatinine level that was closer to normal (<1.2 mg/dL) were more likely (p = 0.001) to have stable or de-escalated oxygen requirements while on NIPPV. Overall, 53.6% of patients who had stable or de-escalated oxygen requirements had a normal serum creatinine level prior to NIPPV initiation compared to 51.3% of patients who had escalated oxygen requirements. The average serum creatinine level for the escalation cohort was 1.77 mg/dL while it was 2.14 mg/dL for the de-escalation cohort, suggesting that the de-escalation cohort had on average a higher serum creatinine level at the time of NIPPV initiation than those who required intubation. Interestingly, creatinine levels significantly decreased in the de-escalation cohort (p = 0.006) while significantly uptrend in the escalation cohort (p <0.001) in patients who had above normal creatinine levels at the time of NIPPV initiation (Figure 2).

Correlating platelet levels with oxygen changes

On average, all patients in this cohort had normal platelet counts. The average platelet count for patients who had an escalation of their oxygen requirements was 241,000/µL prior to initiation on NIPPV, while those who had a de-escalation of their oxygen requirements had an average of 252,000/µL. Although linear regression modeling demonstrated a significant difference between the platelet count means relative to the extent of oxygen requirement changes, the clinical impact itself was not substantial.

## Discussion

The multivariate linear regression models were remarkable for significant association with serum albumin, creatinine, and platelet levels with oxygen escalation and de-escalation over five days. However further analysis showed that the significant association with platelets was not clinically impactful as the starting levels were within normal levels, making it a poor indicator prior to NIPPV initiation. Similarly, serum creatinine was further analyzed which showed a trend toward normalization in the cohort of patients who had de-escalation of their oxygen requirements. However, initial serum creatinine levels prior to NIPPV initiation were elevated for all subgroups making it a poor indicator for the prediction of oxygen escalation or de-escalation. Our results suggested that serum albumin was predictive for oxygen changes in pre-intubated patients infected with SARS-CoV-2 prior to NIPPV initiation, such that patients who were likely to improve while on NIPPV had on average normal serum albumin levels. Albumin, as a negative acute-phase reactant, would decrease in serum levels as systemic inflammation worsened. As such, a trend toward normalizing albumin levels signified improved prognostic outcomes.

Several studies have examined biomarkers to evaluate the severity of SARS-CoV-2. Zeng et al. conducted a meta-analysis of 16 studies and determined that low levels of CRP, procalcitonin, interleukin-6 (IL-6), ESR, serum albumin, and serum ferritin were associated with the non-severe SARS-CoV-2 patient cohort [7]. However, no study has examined biomarkers with oxygen escalation or de-escalation directly. To our knowledge, this study is the first to do so, with importance placed on serum albumin levels.

Low serum albumin levels have frequently been reported in many SARS-CoV-2 studies, but with little understanding of its utility. During the original occurrence of the virus, low serum albumin levels were demonstrated in 6-98% of admitted patients [8] and were associated with poor outcomes including mortality [9]. In another study that reviewed 109 patients with hypoalbuminemia, those with higher albumin levels at the time of admission were less likely to develop acute respiratory distress syndrome (ARDS), be admitted to the intensive care unit, and be readmitted within 90 days after discharge [10]. Low serum albumin levels at the time of admission were associated with a higher risk of death, such that the risk increased by 25% for every 5 g/L decrease in serum albumin level [11]. Albumin may also have a better prognostic value than CRP when determining the course of mild-to-severe ARDS after developing new-onset fever [12].

Recently, a newly devised multivariable predictive model called the AIDA score was validated in a cohort of 304 patients with SARS-CoV-2 admitted to the intensive care unit in Serbia [13]. This score assigned 5 points for patients with low serum albumin levels and 1 point for elevated IL-6 levels, D-dimer levels, and age of >65 years. A risk score >2 was defined as “high risk,” suggesting that low serum albumin levels alone were sufficiently concerning for the likelihood of mortality in patients admitted to the intensive care unit.

Limitations of this study include the inability to account for other confounders due to the limited information available from the data extraction during a high-demand setting, correlation with outcomes after five days, and an association with baseline albumin levels at admission. However, despite the limitations of this study, we propose that serum albumin level may be a good predictor for oxygen escalation in pre-intubated patients. Low serum albumin poses the risk for quick escalation of oxygen supplementation and may help identify at-risk patients, especially in resource-limited settings.

## Conclusions

We conclude that serum albumin level is a good predictor for oxygen escalation and de-escalation in pre-intubated patients with SARS-CoV-2 and can be utilized for risk stratification and resource allocation in high-demand settings. Albumin was predictive for oxygen changes in pre-intubated patients infected with SARS-CoV-2 prior to NIPPV initiation, such that patients who were likely to improve while on NIPPV had on average normal serum albumin levels. Furthermore, a trend toward normalizing albumin levels while on NIPPV also signified improved prognostic outcomes. Risk stratification using albumin may assist in distributing resources in high-demand settings.
